# HSP90AA1-mediated autophagy promotes drug resistance in osteosarcoma

**DOI:** 10.1186/s13046-018-0880-6

**Published:** 2018-08-28

**Authors:** Xin Xiao, Wei Wang, Yuqian Li, Di Yang, Xiaokang Li, Chao Shen, Yan Liu, Xianzhu Ke, Shuo Guo, Zheng Guo

**Affiliations:** 1 Department of Orthopedics, Xijing Hospital, Fourth Military Medical University, Xi’an, Shaanxi 710032 People’s Republic of China; 2 Department of Immunology, State Key Laboratory of Cancer Biology, Fourth Military Medical University, Xi’an, Shaanxi 710032 People’s Republic of China; 3 Department of Neurosurgery, Tangdu Hospital, Fourth Military Medical University, Xi’an, 710038 Shaanxi China; 4 Department of Orthopedics, Navy General Hospital, Beijing, China; 5 State Key Laboratory of Military Stomatology & National Clinical Research Center for Oral Diseases & Shaanxi Key Laboratory of Stomatology, Department of Prosthodontics, School of Stomatology, Fourth Military Medical University, Xi’an, China; 6 Department of Orthopedics, Hubei Cancer Hospital, Wuhan, China

**Keywords:** Autophagy, HSP90AA1, Chemoresistance, Apoptosis, Osteosarcoma

## Abstract

**Background:**

Osteosarcoma is the most common primary bone tumor in children and adolescents. Unfortunately, osteosarcoma treatments often fail due to the development of chemoresistance, of which the underlying molecular mechanisms still remain unclear. In this study, we demonstrated that HSP90AA1 gene is responsible for drug resistance in osteosarcoma through an autophagy-related mechanism.

**Methods:**

shRNAs were transfected into osteosarcoma cells for knockdown of HSP90AA1 gene. Stable HSP90AA1 overexpressing osteosarcoma cell lines were obtained by lentivirus infection. mRNA and protein expressions of HSP90AA1 in osteosarcoma cells were tested by quantitative real-time PCR and western blot, respectively. Autophagy of osteosarcoma cells was detected by western blot of LC3, transmission electron microscopy and fluorescence microscope. mRFP-GFP-LC3 lentiviral transfection was also performed to detect autophagic flux. NOD/SCID mices were inoculated with MG-63 tumor cells transfected with HSP90AA1 specific shRNA. TUNEL and LC3 staining were performed to detect apoptosis and autophagy of resected tumor tissues.

**Results:**

Doxorubicin, cisplatin, and methotrexate, which are commonly used in chemotherapy, each induced HSP90AA1 upregulation in human osteosarcoma cells. Suppression of HSP90AA1 restored the sensitivity of osteosarcoma cells to chemotherapy both in vivo and in vitro. Mechanism study indicated that autophagy is responsible for the chemoresistance in osteosarcoma cells. HSP90AA1 increased drug resistance by inducing autophagy and inhibiting apoptosis. Suppression of HSP90AA1 diminished autophagic protection in response to chemotherapy in osteosarcoma cells. Moreover, HSP90AA1 promotes autophagy through PI3K/Akt/mTOR pathway and inhibits apoptosis through JNK/P38 pathway.

**Conclusion:**

We showed that chemotherapy agents can induce HSP90AA1 expression in osteosarcoma cells. And HSP90AA1, acting as an important regulator of autophagy, is a critical factor in the development of osteosarcoma chemoresistance both in vitro and in vivo. HSP90AA1 provides a novel therapeutic target for improving osteosarcoma treatment.

**Electronic supplementary material:**

The online version of this article (10.1186/s13046-018-0880-6) contains supplementary material, which is available to authorized users.

## Background

Osteosarcoma is the most common primary malignant tumor of bone that occurs mainly in childhood and adolescence [1]. Treatment with a combination of neoadjuvant chemotherapy and surgery has improved the survival rate of osteosarcoma patients [2, 3]. Doxorubicin, cisplatin and methotrexate are commonly used chemotherapy drugs in osteosarcoma treatment [4, 5]. However, the survival rate has remained largely unchanged during the last three decades owing to patients’ poor respond to these drugs. Even though additional doses or drugs are used, these patients will still undergo local recurrence and metastasis, reducing the 5-year-survival rates to only 20% [6, 7]. For this poor prognosis, drug resistance is the main reason. Thus, to develop novel therapies and to finally improve the prognosis of osteosarcoma patients, it is very important to thoroughly understand the molecular mechanisms of the chemotherapy resistance occurred in osteosarcoma cells.

Autophagy, a fundamental lysosomal process that participates in stress tolerance, is involved in many physiological and pathological conditions, such as intracellular recycling, nutrition starvation and, importantly, chemotherapy [8, 9]. By autophagy, impaired proteins and organelles are degraded through delivery to lysosomes and then are recycled to maintain homeostasis and prevent the accumulation of damaged cell fragments, which may lead to cell death [10–12]. Thus, autophagy may serve as a protective mechanism against cell stress and confer to chemoresistance in many types of tumor cells [13–15]. However, the relationship between autophagy and apoptosis, the detailed mechanism and significance of autophagy in osteosarcoma chemoresistance remains largely unknown.

Drug resistance is a multi-factor involved process that is also mediated by cellular stress response to the tumor microenvironment [16]. Heat shock proteins (HSPs) are characterized as highly conserved chaperone proteins which play an important role in cell survival. It has been found that HSPs are responsible for many cytoprotective mechanisms especially under stress conditions [17, 18]. The expressions of HSPs are upregulated in a wide range of tumors upon cell stress and are closely associated with resistance to therapy [19]. Moreover, it has also been discovered that HSPs, such as HSP27, can regulate autophagy [20, 21]. HSP90AA1 belongs to HSP90 family which is one of the most important heat shock protein. It has been reported that HSP90 is a potential molecular target in cancer therapy [22]. Recently, studies found that HSP90AA1 is expressed extracellularly and involved in tumor progression [23, 24] and cancer cell invasion [25]. However, little is known about the role of HSP90AA1 in autophagy and in osteosarcoma drug resistance. Therefore, it would be important to determine the role of HSP90AA1 in osteosarcoma cells with a particular focus on autophagy and its potential effects on the drug resistance. The underlying molecular mechanism of HSP90AA1-mediated autophagy in osteosarcoma chemotherapy also needs to be explored.

In the present study, we found that HSP90AA1 is upregulated during chemotherapy and the autophagy mediated by it contributes to chemotherapy resistance in osteosarcoma both in vivo and in vitro. HSP90AA1 promotes autophagy and inhibits apoptosis through PI3K/Akt/mTOR pathway and JNK/P38 pathway, respectively. Our findings provide novel therapeutic target for the treatment of osteosarcoma.

## Methods

### Cell culture and reagents

Human osteosarcoma cell lines MG-63, Saos-2 and U-2 OS were purchased from the Cell Bank of Chinese Academy of Medical Science (Shanghai, China) and cultured in McCoy’s 5a medium (Gibco, Los Angeles, CA) containing 10% fetal bovine serum (Gibco) and ampicillin and streptomycin at 37 °C in a humidified atmosphere of 95% air and 5% CO2. All cell lines were used within 20 passages. The antibody against HSP90AA1was obtained from Proteintech. The antibodies against LC3, p62, cleaved PARP, Akt, p-Akt, mTOR, p-mTOR, JNK, p-JNK, p38, p-p38 and actin were obtained from Cell Signaling Technology. Cisplatin, doxorubicin, methotrexate, bafilomycin A1, rapamycin, LY294002 and 3-methyladenine were purchased from Sigma Aldrich (St. Louis, MO, USA).

### Cell transfection

Control/HSP90AA1 shRNAs were obtained from GenePharma (Shanghai, China) and were transfected into osteosarcoma cells using the Lipofectamine 3000 Transfection Reagent (Invitrogen, Carlsbad, NM) at final concentration of 50 nM for 48 h according the manufacturer’s instructions.

Cells were transfected with a LC3 plasmid (Dongao Biosciences, China). After 48 h, cells were treated with rapamycin or LY294002 for additional 24 h. Then cells were fixed in 3.7% formaldehyde and LC3-II-positive punctate pattern was observed under fluorescence microscope. Numbers of autophagosomes were counted by using the Image J program.

### HSP90AA1 overexpressing cell line establishment

A lentivirus carrying HSP90AA1 gene was constructed by Hanbio Co. LTD (Shanghai, China). MG-63 and U-2 OS cells were seeded in 12-well plate and then infected with the lentivirus according to protocols as recommended by the manufacturer. After 24 h, the medium was replaced with complete medium. In order to obtain a stable HSP90AA1 overexpressing cell line, the lentivirus infected cells were selected by incubation with 2μg/ml of puromycin. The expression of HSP90AA1 in MG-63 and U-2 OS cell lines stably infected with a lentivirus was examined by Western blot.

### Cell viability

Cell viability was evaluated by the Cell Counting Kit-8 (CCK-8) test as previously described [26]. MG-63, Saos-2 and U-2 OS cells were seeded into 96-well plates. After adhesion, the cells were pretreated with or without 3-MA (5 mM) for 2 h, and then treated with cisplatin, doxorubicin, methotrexate for the indicated concentrations and time-points. Then 10 μl of CCK-8 reagent was added to each well and cultured at 37̊C for 1 h. The relative number of surviving cells was determined by measuring the optical density (O.D.) of the cell lysates at 450 nm.

### Apoptosis assay

The cells were harvested in 0.25% trypsin and washed twice with PBS. The degree of apoptosis in cells was assessed using the Annexin V-PE/propidium iodide (PI) apoptosis detection kit (BD, Shanghai, China) by flow cytometric analysis. The degree of apoptosis in tissue was assessed with the TUNEL kit (Roche) according to the manufacturer’s instructions. In addition, western blot analyses for c-PARP and analysis of caspase 3 activity by Colorimetric Caspase 3 Assay Kit (Merck) after various treatments were performed.

### Quantitative real-time PCR

Total RNA was extracted from cells using TRIzol (Invitrogen). The quantitative real-time PCR (qRT-PCR) experiments were performed using SYBR-Green reagents (Takara Bio Inc., Shiga, Japan) with specific primers for HSP90AA1 (5’-TATAAGGCAGGCGCGGGGGT-3′ and reverse primer, 5′- TGCACCAGCCTGCAAAGCTTCC-3′) and glyceraldehyde-3-phosphate dehydrogenase (GAPDH forward primer, 5’-GAAGGTGAAGGTCGGAGTC-3′ and reverse primer, 5’-GAAGATGGTGATGGGATTTC-3′). Each sample was run in triplicate and the control group was set as 1.

### Western blot analysis

Cells were harvested and lysed in radioimmune precipitation assay (RIPA) buffer, and then the protein concentrations were determined using a BCA kit (Thermo Fisher Scientific). Equal amounts of cell lysates were resolved by SDS-PAGE and transferred to PVDF membranes. The membranes were blocked with 5% skimmed milk, and then incubated overnight at 4̊C with primary antibodies against LC3, p62 and c-PARP (1:1000) followed by incubation with polyclonal HRP-conjugated secondary antibodies (1:2000) for 1 h at room temperature. The membranes were visualized by enhanced chemiluminescence. Actin protein was used as a loading control.

### Analysis of autophagic flux

To monitor the autophagic flux, mRFP-GFP-LC3 lentivirus (Hanbio Co. LTD) transfection was used through marking and tracking LC3. Osteosarcoma cells were transfected with tandem fluorescent mRFP-GFP-tagged adenovirus for 48 h following the manufacturer’s instruction and then treated with cisplatin and doxorubicin for additional 24 h. The images were acquired using confocal fluorescence microscopy. Yellow (merge of GFP signal and RFP signal) puncta represented early autophagosomes, while red (RFP signal alone) puncta indicated late autolysosomes. Autophagic flux was evaluated by the colour change of GFP/mRFP.

### Transmission electron microscopy

Cells were washed with 0.1 cacodylate buffer (pH 7.4) and fixed with a solution containing 3% glutaraldehyde plus 2% paraformaldehyde in PBS. Subsequently, the rest of the procedure was conducted using the standard protocol. The thin sections were stained with uranyl acetate and lead citrate for observation under Zeiss Transmission Electron Microscope as previously described [27].

### Mice xenograft models

All animal experiments strictly followed the guidelines of the Fourth Military Medical University Committee on Animal Care. 5 × 10^6^ MG-63 cells transfected with control or HSP90AA1 shRNA were suspended in PBS and were injected subcutaneously to the right of the dorsal midline in NOD/SCID mice (the Fourth Military Medical University, Shaanxi, China) as previously described [28]. Tumor volumes were examined every other day. When the subcutaneous tumor size had reached a diameter of approximately 3 mm, docetaxel was administered via intraperitoneal injection at a dose of 5 mg/kg twice a week. All mice were killed after 4 weeks. The primary tumors were excised and analyzed by immunofluorescence staining of LC3 and TUNEL staining. Tumor volumes were calculated using the formula: length×width^2^/2(mm3).

### Statistical analyses

All results were confirmed in at least three independent experiments and all data were presented as mean ± SD. Two-sided student’s t-tests or analysis of variance (ANOVA) tests were used to assess statistically significant differences. Values of *p* < 0.05 were considered as statistically significant.

## Results

### Chemotherapy agents promote HSP90AA1 expression in osteosarcoma cells

**Fig. 1:**
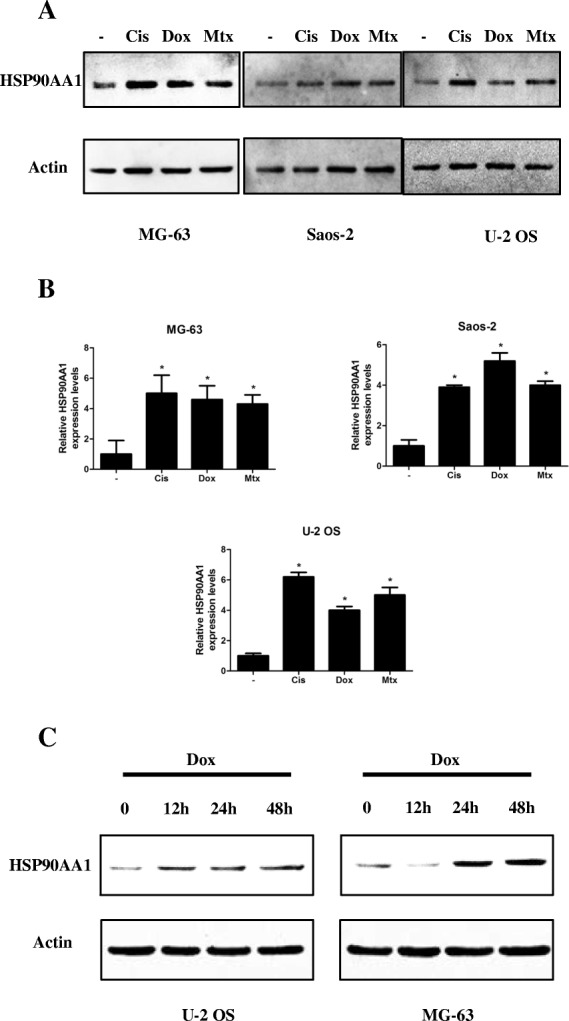
Chemotherapy agents promote HSP90AA1 expression in osteosarcoma cells. **a** and **b**, MG-63, Saos-2 and U-2 OS cells were treated with Cis (20 μmol/L), Dox (0.2 μg/mL), and Mtx (50 μmol/L) for 24 h. Whole cell lysates were subjected to western blot analysis for HSP90AA1 expression (**a**) and HSP90AA1 mRNA level was assessed by quantitative real-time PCR (**b**; *n* = 3; *, *p* < 0.05 versus untreated group). **c** MG-63 and U-2 OS cells were treated with Dox (0.2 μg/mL) for 12 to 48 h and then HSP90AA1 protein level was assessed by Western blot

We analyzed the expression of HSP90AA1 in osteosarcoma cell lines exposed to chemotherapy agents cisplatin (Cis), doxorubicin (Dox), and methotrexate (Mtx). These drugs significantly increased the expression of HSP90AA1 in the MG-63, Saos-2 and U-2 OS cell lines (Fig. 1a and Additional file 1: Figure S1a). Consistent with the western blot results, the mRNA expression level of HSP90AA1 was also increased after treatment with these drugs (Fig. 1b). Moreover, following exposure to doxorubicin, this effect was in a time dependent manner. The HSP90AA1 expression was elevated at 12 h in U-2 OS cells and at 24 h in MG-63 cells and in both of the cells lines, the level of HSP90AA1 kept rising until 48 h (Fig. 1c and Additional file 1c: Figure S1b). These results indicate that HSP90AA1 is upregulated during chemotherapy in osteosarcoma cells.

### HSP90AA1 reduces sensitivity of osteosarcoma cells to chemotherapy by decreasing apoptosis

**Fig. 2:**
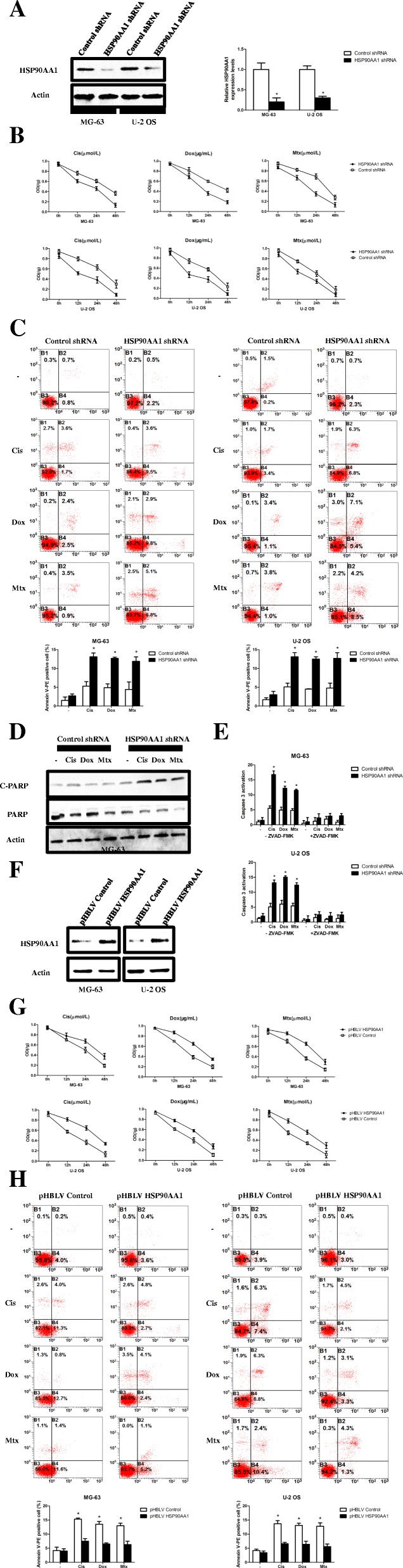
HSP90AA1 reduces osteosarcoma cells sensitivity to chemotherapy by decreasing apoptosis. **a** MG-63 and U-2 OS osteosarcoma cells were transfected with control shRNA and HSP90AA1 shRNA for 48 h. Then the expression levels of HSP90AA1 were analyzed by Western blot and quantitative real-time PCR (*n* = 3; *, *p* < 0.05 versus control shRNA group). **b**, **c** and **d**, after transfection with control shRNA and HSP90AA1 shRNA, MG-63 and U-2 OS cells were treated with Cis (20 μmol/L), Dox (0.2 μg/mL) and Mtx (50 μmol/L) for 24 h. Then cell viability was determined at 12, 24 and 48 h by CCK-8 assay (**b**). Apoptosis was analyzed at 24 h by flow cytometric analysis of Annexin V-PE/PI staining (**c**; *n* = 3; *, *p* < 0.05 versus control shRNA group) and both cleaved and total PARP in MG-63 cells were analyzed by Western blot (**d**). **e** After transfection with control shRNA and HSP90AA1 shRNA, MG-63 and U-2 OS cells were exposed to Cis (20 μmol/L), Dox (0.2 μg/mL) and Mtx (50 μmol/L) for 24 h in the presence or absence of ZVAD-FMK (20 μmol/L). Then apoptosis was evaluated by Colorimetric Caspase 3 Assay Kit (*n* = 3; *, *p* < 0.05 versus control shRNA group). **f** MG-63 and U-2 OS cells were infected with control (pHBLV control) and HSP90AA1-expressing lentiviruses (pHBLV HSP90AA1). The protein level of HSP90AA1 was assayed by Western blot. G and H, HSP90AA1-overexpressing MG-63 and U-2 OS cells were treated with Cis (20 μmol/L), Dox (0.2 μg/mL) and Mtx (50 μmol/L) for 24 h. Cell viability was determined at 12, 24 and 48 h by CCK-8 assay (**g**) and apoptosis was analyzed at 24 h by measuring Annexin V-PE positive cells by flowcytometry (**h**; *n* = 3; *, *p* < 0.05 versus control shRNA group)

To explore the potential role of HSP90AA1 in osteosarcoma cells’ sensitivity to chemotherapy, HSP90AA1 shRNA was transfected into MG-63 and U-2 OS cells. We found that HSP90AA1 shRNA transfection markedly decreased HSP90AA1 mRNA and protein expressions in these cells (Fig. 2a and Additional file 1: Figure S1c). Knockdown of HSP90AA1 inhibited cell proliferation (Fig. 2b) and enhanced the cellular response to cisplatin, docetaxel and methotrexate in MG-63 and U-2 OS cells. High levels of apoptotic cell death were found, as shown by an increase of Annexin V-PE positive cells (Fig. 2c) and an increase level of cleaved PARP (Fig. 2d and Additional file 1: Figure S1d) in HSP90AA1 knockdown cells compared with control shRNA treated groups. At the end of the 24 h treatment with these chemotherapy drugs, the proapoptotic protein caspase 3 was activated to a greater extent after HSP90AA1 knockdown in MG-63 and U-2 OS cells. Moreover, treatment with the apoptosis inhibitor ZVAD-FMK reversed the activation of caspase 3 activity (Fig. 2e).

**Fig. 3:**
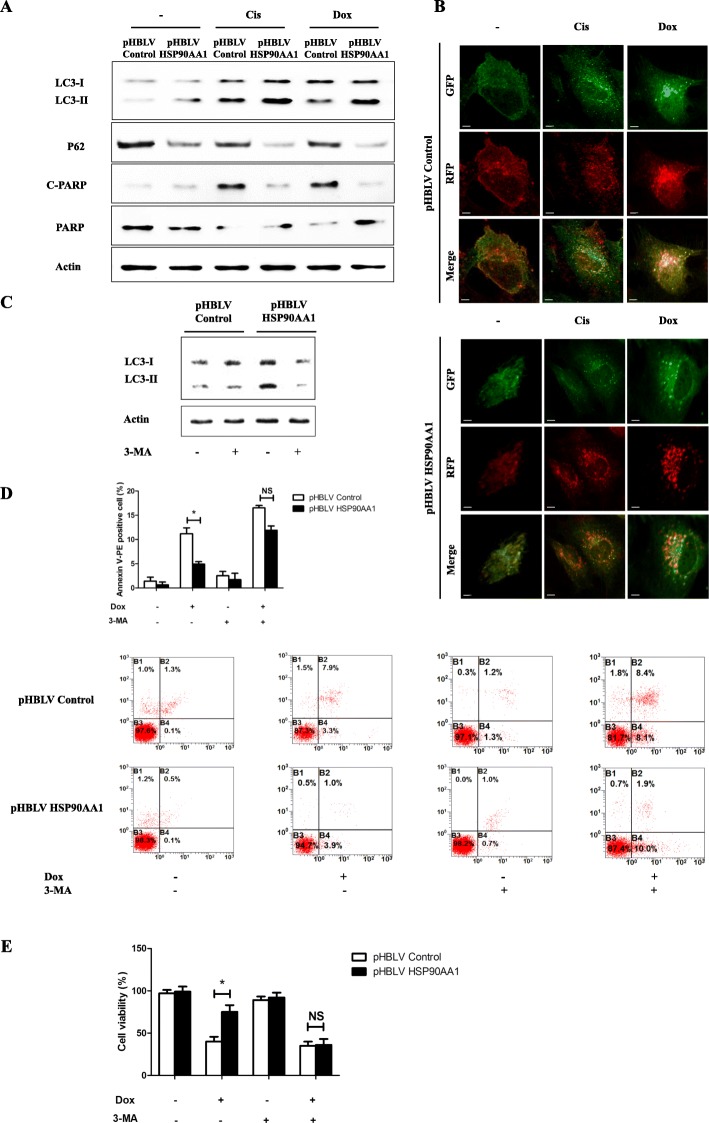
Autophagy mediates HSP90AA1-induced sensitivity of osteosarcoma cells to chemotherapy. **a** and **b** HSP90AA1-overexpressing MG-63 cells were treated with Cis (20 μmol/L) or Dox (0.2 μg/mL) for 24 h. The protein levels of LC3, p62 and both cleaved and total PARP were assayed by Western blot (**a**). Autophagic flux were analyzed by mRFP-GFP-LC3 construct (**b**). **c** HSP90AA1-overexpressing MG-63 cells were treated with 3-methyladenine (3-MA, 5 mM). Autophagy activity indicated by LC3 levels was analyzed by western blot. **d** and **e** HSP90AA1-overexpressing MG-63 cells were treated with Dox (0.2 μg/mL) for 24 h with or without 3-MA (5 mM 2 h). Apoptosis was assessed by analysis of Annexin V-PE/PI staining (**d**, *n* = 3; *, *p* < 0.05 versus pHBLV control group) and proliferation was analyzed by CCK-8 assay (**e**, *n* = 3; *, *p* < 0.05 versus pHBLV control group). NS, not significant

To further clarify the role of HSP90AA1 in osteosarcoma cells after chemotherapy, a lentivirus carrying HSP90AA1 gene was constructed and infected into MG-63 and U-2 OS osteosarcoma cells (Fig. 2f and Additional file 1: Figure S1e). Overexpression of HSP90AA1 rendered them resistant to apoptosis induced by cisplatin, doxorubicin and methotrexate, as indicated by an increase in the cell proliferation rate (Fig. 2g), a decrease in the number of Annexin V-PE positive cells (Fig. 2h), and a decrease in the level of cleaved PARP (Fig. 3a and Additional file 1: Figure S1f) compared with pHBLV control treated cells, confirming an antiapoptotic role of HSP90AA1 in osteosarcoma cells. These results demonstrate that HSP90AA1 increases the resistance of osteosarcoma cells to chemotherapy by decreasing the apoptosis.

### HSP90AA1 promotes autophagy during chemotherapy in osteosarcoma cells

Sometimes autophagy and apoptosis can be triggered by common upstream signals, leading to initiation of both processes simultaneously [29]. To determine whether HSP90AA1 regulates autophagy, we evaluated autophagy activity in osteosarcoma cells after treatment with chemotherapy agents. Overexpression of HSP90AA1 in MG-63 cells led to an increase in the level of LC3-II and decrease in the amount of p62 after cisplatin and doxorubicin treatment (Fig. 3a). Then the autophagic flux was also detected by mRFP-GFP-LC3 lentiviral transfection in pHBLV Control/HSP90AA1 MG-63 cells. Chemotherapy treatment increased the level of autophagosomes (yellow dots in merged images) in the control group, while the level of autophagosome-lysosomes (red dots in merged images after the GFP quenched) were significantly increased after overexpression of HSP90AA1, meaning that HSP90AA1 is able to promote the autophagic flux in chemotherapy agents treated osteosarcoma cells (Fig. 3b). Next, to explore whether autophagy influences the effects of HSP90AA1-mediated resistance to apoptosis after treatment with chemotherapy drugs, we treated pHBLV Control/HSP90AA1 MG-63 cells with an autophagy inhibitor 3-methyladenine (3-MA). The 3-MA efficiently inhibited HSP90AA1 overexpression-induced activation of autophagy (Fig. 3c and Additional file 1: Figure S1g). Moreover, the 3-MA also leads to an increase of apoptosis (Fig. 3d) and decrease in the proliferation rate (Fig. 3e) in pHBLV Control/HSP90AA1 MG-63 cells after treatment with doxorubicin, which indicates the autophagy inhibitor reversed the HSP90AA1-induced protection against chemotherapy. These findings demonstrate that autophagy is required for HSP90AA1-mediated antiapoptotic effect.

**Fig. 4:**
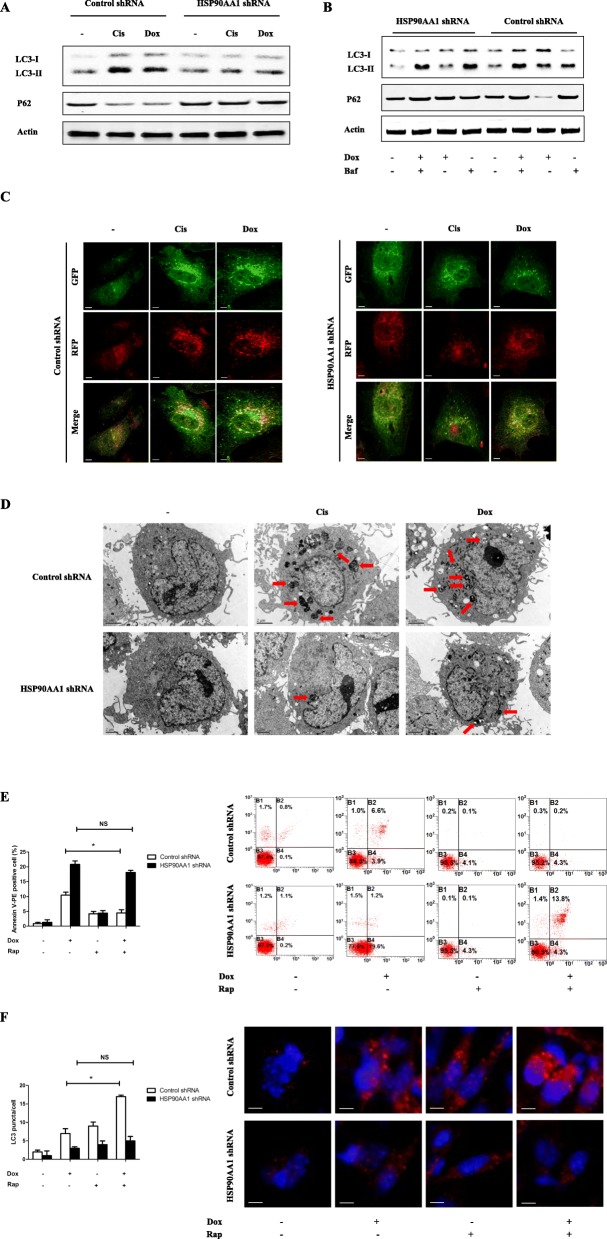
Knockdown of HSP90AA1 inhibits autophagy during chemotherapy in osteosarcoma cells. **a** MG-63 osteosarcoma cells were transfected with control shRNA/HSP90AA1 shRNA for 48 h and then were treated with Cis (20 μmol/L) or Dox (0.2 μg/mL) for 24 h. The protein levels of LC3 and p62 were assessed by Western blot. **b** Control shRNA/HSP90AA1 shRNA transfected MG-63 cells were treated with Dox (0.2 μg/mL) for 24 h with or without bafilomycin A1 (100 nmol/L). LC3 and p62 expression were analyzed by Western blot. **c** and **d** control shRNA/HSP90AA1 shRNA transfected MG-63 cells were treated with Cis (20 μmol/L) or Dox (0.2 μg/mL) for 24 h. Then autophagic flux were analyzed by mRFP-GFP-LC3 construct (**c**) and transmission electron microscopy (**d**), autophagosome-like structures were indicated (red arrows). **e** and **f** Control shRNA/HSP90AA1 shRNA transfected MG-63 cells were pretreated with rapamycin (100 nmol/L) for 6 h and then were exposed to Dox (0.2 μg/mL) for an additional 24 h. Apoptosis was analyzed by measuring Annexin V-PE/PI positive cells by flow cytometric (**e**, *n* = 3; *, *p* < 0.05, versus rapamycin untreated group). Autophagy was determined by measuring LC3 puncta formation (**f**, *n* = 3; *, *p* < 0.05 versus rapamycin untreated group). NS, not significant

To further explore whether HSP90AA1 regulates autophagy in osteosarcoma cells, the shRNA against HSP90AA1 was introduced. Western blot analyses revealed that knockdown of HSP90AA1 inhibited chemotherapy-induced the formation of LC3-II and the degradation of P62 (Fig. 4a and Additional file 1: Figure S1h). Upon the formation of autophagosomes, recruitment of LC3 can be seen in the autophagosomal membrane. And LC3-II was observed to accumulate in the presence of bafilomycin A1, an autophagy-lysosomal inhibitor (Fig. 4b and Additional file 1: Figure S1i). Meanwhile, compared with the control group, the autophagic flux was also blocked in HSP90AA1 shRNA transfected cells after chemotherapy agents treatment (Fig. 4c). As displayed in Fig. 4d, HSP90AA1 shRNA brought MG-63 cells decreased autophagy activity during chemotherapy compared with control shRNA treated cells as measured by ultra-structural analysis of autophagosomes using transmission electron microscopy. Taken together, these findings suggest that HSP90AA1 promotes autophagy in osteosarcoma cells. Rapamycin, which induces autophagy by inhibiting mTOR protein, protected HSP90AA1 wild-type cells against apoptosis induced by doxorubicin, but conferred less protection in HSP90AA1 knockdown cells (Fig. 4e). This effect was attributed to the diminished autophagic capacity caused by HSP90AA1 knockdown (Fig. 4f). These results demonstrate that HSP90AA1 is an important regulator of autophagy-mediated cell survival.

### HSP90AA1 promotes autophagy and inhibits apoptosis through PI3K/Akt/mTOR pathway and JNK/P38 pathway respectively

**Fig. 5:**
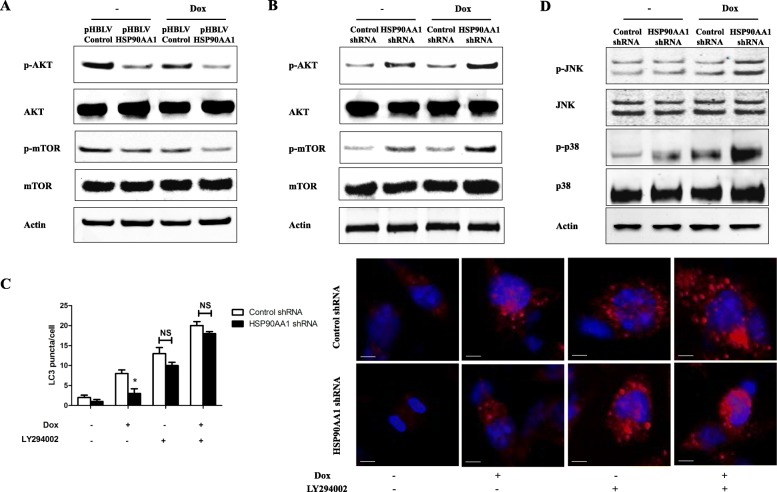
HSP90AA1 promotes autophagy and inhibits apoptosis through PI3K/Akt/mTOR pathway and JNK/P38 pathway respectively. **a** and **b** HSP90AA1-overexpressing MG-63 cells and HSP90AA1 shRNA transfected MG-63 cells were treated with Dox (0.2 μg/mL) for 24 h. Total cell lysates were subjected to western blot analysis of key proteins in the PI3K/Akt/mTOR pathway. **c** Control shRNA/HSP90AA1 shRNA transfected MG-63 cells were treated with Dox (0.2 μg/mL) for 24 h with or without LY294002 (10 μM). Then autophagy was analyzed by measuring LC3 puncta formation (*n* = 3; *, *p* < 0.05 versus control shRNA group). NS, not significant. **d** MG-63 cells transfected with control/HSP90AA1 shRNA for 48 h and were treated with Dox (0.2 μg/mL) for 24 h. Total cell lysates were subjected to western blot analysis of key proteins in JNK/p38 pathway

Previous studies has demonstrated that PI3K/Akt/mTOR pathway is associated with drug-induced autophagy in cancer cells [30, 31]. In osteosarcoma drug resistance, few work has been done to show inhibition of autophagy by PI3K/Akt/mTOR pathway. Thus, we investigated the role of the PI3K/Akt/mTOR pathway in HSP90AA1 induced autophagy by western blot analysis. MG-63 cells infected with pHBLV Control/HSP90A1 lentivirus were treated with doxorubicin. We observed that overexpression of HSP90AA1 inhibited the PI3K/Akt/mTOR pathway by significantly decreasing the expressions of phosphorylation of Akt and mTOR. This inhibition effect was more dramatic after treatment with doxorubicin (Fig. 5a and Additional file 1: Figure S1j). Knockdown of HSP90AA1 markedly increased Akt and mTOR phosphorylation in MG-63 cells especially after treatment with doxorubicin (Fig. 5b and Additional file 1: Figure S1k). Moreover, the PI3K inhibitor LY294002 was applied in MG-63 cells before doxorubicin treatment. The results showed that LY294002 treatment restored the autophagy inhibited by HSP90AA1 shRNA transfection (Fig. 5c). These results demonstrated that the HSP90AA1 could promote chemotherapy-induced autophagy through inhibiting the PI3K/Akt/mTOR signaling pathway in osteosarcoma cells.

The p38 and JNK pathway plays an important role in regulating cell apoptosis. To confirm the effects of the JNK/p38 pathway in HSP90AA1-mediated inhibition on apoptosis, we transfected control/HSP90AA1 shRNA into MG-63 cells and then the cells were exposed to doxorubicin. The results showed that knockdown of HSP90AA1 markedly increased the protein expression levels of p-JNK and p-p38 kinase in response to doxorubicin (Fig. 5d and Additional file 1: Figure S1l). Moreover, the JNK inhibitor SP600125 and p38 inhibitor SB203580 were applied in MG-63 cells before doxorubicin treatment. The results showed that, in the treatment of doxorubicin, SP600125 and SB203580 treatments both inhibited the cell apoptosis increased by HSP90AA1 knockdown (Additional file 2: Figure S2). These results implied that HSP90AA1 inhibited cell apoptosis through deactivation of JNK and p38 kinase pathways.

### Suppression of HSP90AA1 increases sensitivity to chemotherapy in vivo

**Fig. 6:**
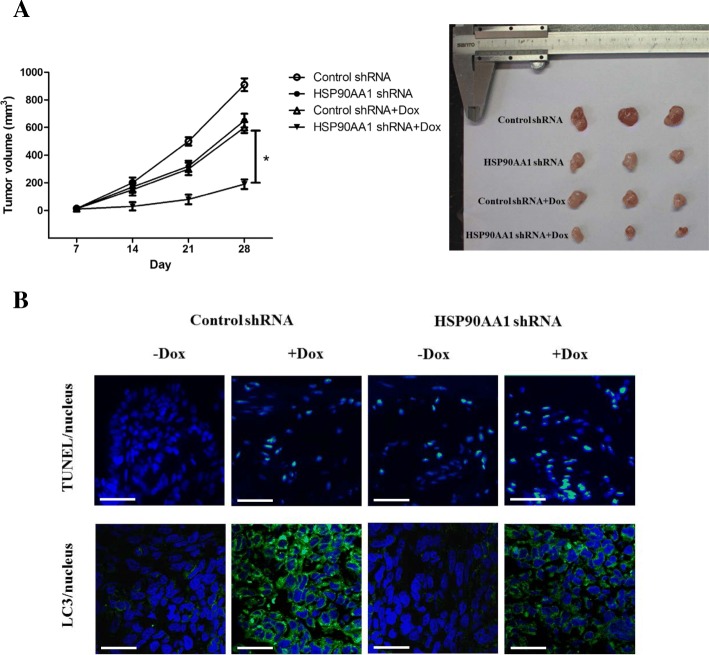
Suppression of HSP90AA1 increases sensitivity to chemotherapy in vivo. **a** NOD/SCID mice were inoculated with control shRNA/HSP90AA1 shRNA transfected MG-63 cells. At the beginning at day 7, docetaxel (5 mg/kg) was administered via intraperitoneal injection. **a** tumor volumes were calculated for 28 days (*n* = 3; *p < 0.05). Representative photographs of tumors formed at 4 weeks are shown. **b** Apoptosis and autophagy in tumor tissues were detected by TUNEL and LC3 stain respectively after insection of tumor samples

To explore the effect of knockdown of HSP90AA1 on chemosensitivity of osteosarcoma cells in vivo, the NOD/SCID mice was inoculated with MG-63 tumor cells transfected with HSP90AA1 shRNA. Mice was then treated with doxorubicin beginning at day 7. Tumors derived from HSP90AA1 knockdown cells grew more slowly compared with those derived from control shRNA transfected cells after doxorubicin treatment (Fig. 6a). By TUNEL staining, we found that the resected tumor tissues with HSP90AA1 shRNA transfection showed higher apoptosis than those with control shRNA transfection in response to chemotherapy (Fig. 6b). We also observed a decreased autophagy in HSP90AA1 shRNA group in response to docetaxel in comparison with control group (Fig. 6b). These findings support an important role of HSP90AA1 in modulating drug resistance in osteosarcoma cells in vivo.

## Discussion

Doxorubicin, cisplatin and methotrexate are the standard chemotherapeutic drugs used in osteosarcoma treatment. However, many patients develop resistance to these drugs and this acquired resistance to chemotherapy of osteosarcoma cells remains a big challenge for overall survival. A variety of mechanisms contributing to drug resistance have been well established such as DNA repair and resistance to apoptosis [32]. Autophagic cell death was previously considered an alternative cell death form due to excessive self-digestion though no apoptosis happens. However, much more evidence indicated that autophagy functions as a pro-survival mechanism, especially when different kinds of stresses related to cell death, such as chemotherapy, are undergoing in cells [33]. Moreover, it has been reported that various chemotherapy drugs can induce autophagic response in cancer cells, which is regarded as a mechanism of chemotherapeutic resistance. Inhibition of autophagy may possibly improve the chemotherapeutic response [33, 34]. However, in osteosarcoma treatment, the key molecules and mechanisms for promoting sensitivity of chemotherapy remains to be elucidated. In this study, consistent with the cytoprotective role of autophagy in cancer cells, we demonstrated for the first time that HSP90AA1-mediated autophagy is a significant contributor to drug resistance in osteosarcoma. Suppression of HSP90AA1 or blockade of autophagy increased the drug sensitivity of osteosarcoma cells. This detailed understanding of autophagy regulated by HSP90AA1 in osteosarcoma might contribute to predicting and overcoming chemoresistance, thereby improving the therapeutic efficacy and overall survival of osteosarcoma patients.

HSP90AA1 is a ubiquitously expressed molecular chaperone that is involved in the stability of mutated and overexpressed signaling proteins that promote the survival of cancer cells [35]. It is upregulated in several cancers, such as leukemia and bladder cancer [23, 36]. Importantly, HSP90AA1 is regarded as essential for malignant transformation and progression. Increased level of HSP90AA1 suggests poor prognosis. A clinical study with 206 gastric cancer patients reported that loss of HSP90AA1 is associated with positive clinical outcomes [37]. Other studies also found that, in tumor biopsies, the absence of HSP90AA1 may serves as a biomarker for favorable outcomes [38, 39]. In addition, it has been reported that HSPs, such as HSP27, regulate autophagy in the condition of cell stress, such as acute injuries. However, whether HSP90AA1 regulates autophagy when osteosarcoma cells subjected to chemotherapy and the effect of HSP90AA1 on drug resistance in osteosarcoma remain unknow. Hence, in order to improve the survival rate of osteosarcoma through overcome chemoresistance, our focus here was the interaction between HSP90AA1 and autophagy.

We firstly observed that cisplatin, doxorubicin and methotrexate promoted the expression of HSP90AA1 in human osteosarcoma cells, suggesting that HSP90AA1 may correlate with chemotherapy resistance. Moreover, we evaluated the association between expression of HSP90AA1 and survival of osteosarcoma cells after anticancer agents treatment. Our findings showed that knockdown of HSP90AA1 by shRNA promoted cell apoptosis and inhibited osteosarcoma growth both in vivo and in vitro. Meanwhile, Overexpression of HSP90AA1 rendered them resistant to apoptosis and increased the cell proliferation rate. These results proved that HSP90AA1 promotes chemoresistance in osteosarcoma cells.

Tumor cells respond to chemotherapy in several ways including initiation of cell death and activation of survival pathways. Previous studies reported that no single anticancer agent can induce cell death by autophagy, [40] indicating the cytoprotective mechanism of autophagy in cancer cells [41]. In this study, we evaluated the autophagy activity in osteosarcoma cells after treatment with chemotherapy and found that knockdown of HSP90AA1 blocked the autophagic flux detected by mRFP-GFP-LC3 construct. A decrease in autophagy-related protein LC3 II and P62 degradation, which were associated with decreased number of membraned autophagosomes, were also found in HSP90AA1 knockdown group. Whereas overexpression of HSP90AA1 results in the contrary findings. Importantly, we found that inhibition of autophagy promotes osteosarcoma cell apoptosis and reverses HSP90AA1-mediated drug resistance. HSP90AA1-induced protection against chemotherapy was reversed when using autophagy inhibitors. These data revealed that autophagy mediated by HSP90AA1 plays a key role in chemotherapy sensitivity. One study showed that HSP90 inhibitor, geldanamycin, induced autophagy in osteosarcoma cells under normal condition, which indicated the potential anti-autophagy role of this protein [42]. In the present study, we found an opposite role of HSP90 on autophagy, but it is from an isoform of this protein. HSP90α is a well-known isoform of HSP90 and is encoded by HSP90AA1 gene. We demonstrated that, under chemotherapy drug stress, inhibition of HSP90AA1 decreased the autophagy of osteosarcoma cells, which indicated the pro-autophagy role of HSP90α. The differences between these two findings may be explained by the following reasons. Firstly, HSP90 and its isoform HSP90α may play different roles even in the same type of cells. Secondly, a transition of the cellular state, from in normal condition to under chemotherapy drug stress, may change the function of the same protein. Studies on different isoforms of protein under different cellular states will help us understand its roles in tumor progression more comprehensively.

**Fig. 7:**
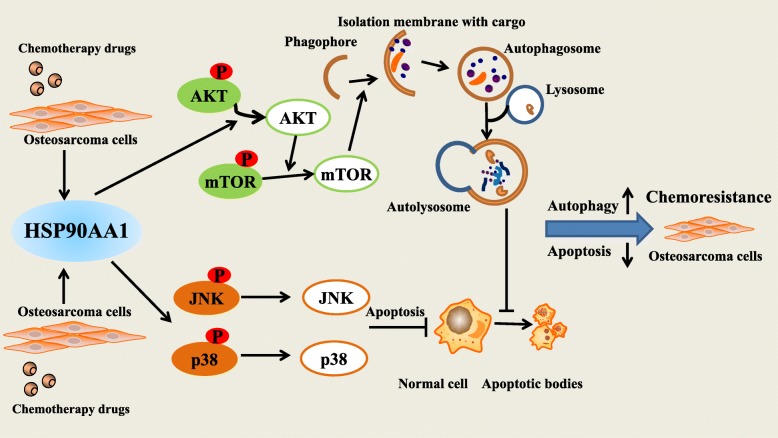
Proposed model of the mechanism of HSP90AA1 gene in osteosarcoma drug resistance. Chemotherapy increases the expression level of HSP90AA1, which leads to disassociation of phosphorylated AKT and JNK/P38. The dephosphorylation of AKT decreases the expressions of phosphorylation of mTOR and promotes autophagy. The dephosphorylation of JNK/P38 results in decreased apoptosis. Moreover, autophagy also can influence HSP90AA1-mediated antiapoptotic effect. Therefore, HSP90AA1 is a critical factor in the development of osteosarcoma chemoresistance

PI3K is a crucial upstream autophagy regulator, and PI3K/AKT signaling is a well characterized pathway that contributes to mTOR activation [43]. PI3K/AKT/mTOR signaling pathway has been demonstrated to play an important role in regulating autophagy in cancer cells [44]. In the present study, overexpression of HSP90AA1 decreased the phosphorylation of Akt and mTOR while knockdown of HSP90AA1 increased the phosphorylation of Akt and mTOR. Furthermore, the autophagy inhibited by knockdown of HSP90AA1 was restored in the presence of the PI3K inhibitor LY294002. These findings proved that inhibition of the PI3K/Akt/mTOR signaling pathway might contribute to the HSP90AA1-induced autophagy in osteosarcoma (Fig. 7). JNKs and p38 were identified to be activated in response to a variety of cellular stress such as chemotherapy, heat shock and DNA damage [45, 46]. In most cases this pathway participates in cell stress-induced alterations through regulating cell apoptosis. In the present study, we observed an increase in phosphorylation of JNK and p38 in HSP90AA1 shRNA transfected osteosarcoma cells. These demonstrated that the HSP90AA1 inhibited apoptosis at least partially through down regulation of the JNK/p38 pathway (Fig. 7).

## Conclusion

In conclusion, here we showed chemotherapy agents induce HSP90AA1 expression in osteosarcoma cells. HSP90AA1 acts as an important regulator of autophagy that leads to inhibited apoptosis and increased drug resistance. HSP90AA1 promotes autophagy and inhibits apoptosis through PI3K/Akt/mTOR pathway and JNK/P38 pathway. We demonstrated that suppression of HSP90AA1 expression significantly increased drug sensitivity of osteosarcoma cells both in vitro and in vivo. These results support an investigation of HSP90AA1 as a potential target for osteosarcoma therapy.

## Additional files

Additional file 1: **Figure S1.** Normalized quantification of all immunoblots in the manuscript. A, The normalized quantification of immunoblots in Fig. 1a. B, The normalized quantification of immunoblots in Fig. 1c. C, The normalized quantification of immunoblots in Fig. 2a. D, The normalized quantification of immunoblots in Fig. 2d. E, The normalized quantification of immunoblots in Fig. 2f. F, The normalized quantification of immunoblots in Fig. 3a. G, The normalized quantification of immunoblots in Fig. 3c. H, The normalized quantification of immunoblots in Fig. 4a. I, The normalized quantification of immunoblots in Fig. 4b. J, The normalized quantification of immunoblots in Fig. 5A. K, The normalized quantification of immunoblots in Fig. 5b. L, The normalized quantification of immunoblots in Fig. 5d. (*n* = 3; *, *p* < 0.05 versus control group). (PDF 304 kb)

Additional file 2: **Figure S2.** JNK or p38 inhibitors decreased the cell apoptosis. A, control shRNA/HSP90AA1 shRNA transfected MG-63 cells were treated with Dox (0.2 μg/mL) for 24 h with or without SP600125. Apoptosis was analyzed by measuring Annexin V-PE/PI positive cells by flow cytometric (*n* = 3; *, *p* < 0.05 versus control shRNA group). B, control shRNA/HSP90AA1 shRNA transfected MG-63 cells were treated with Dox (0.2 μg/mL) for 24 h with or without SB203580. Apoptosis was analyzed by measuring Annexin V-PE/PI positive cells by flow cytometric (*n* = 3; *, *p* < 0.05 versus control shRNA group). NS, not significant. (PDF 88 kb)

## Abbreviations

Cis: Cisplatin
HSP90AA1: Heat shock protein 90 alpha family class A member 1
Dox: Doxorubicin.
Mtx: Methotrexate
3-MA: 3-methyladenine
Rap: Rapamycin
qRT-PCR: quantitative reverse transcription polymerase chain reaction
