# Supplementary figures and images for: HSP90AA1-mediated autophagy promotes drug resistance in osteosarcoma
Source: J Exp Clin Cancer Res. 2018 Aug 28;37:201. doi: 10.1186/s13046-018-0880-6 (PMC6114771; doi:10.1186/s13046-018-0880-6)

A

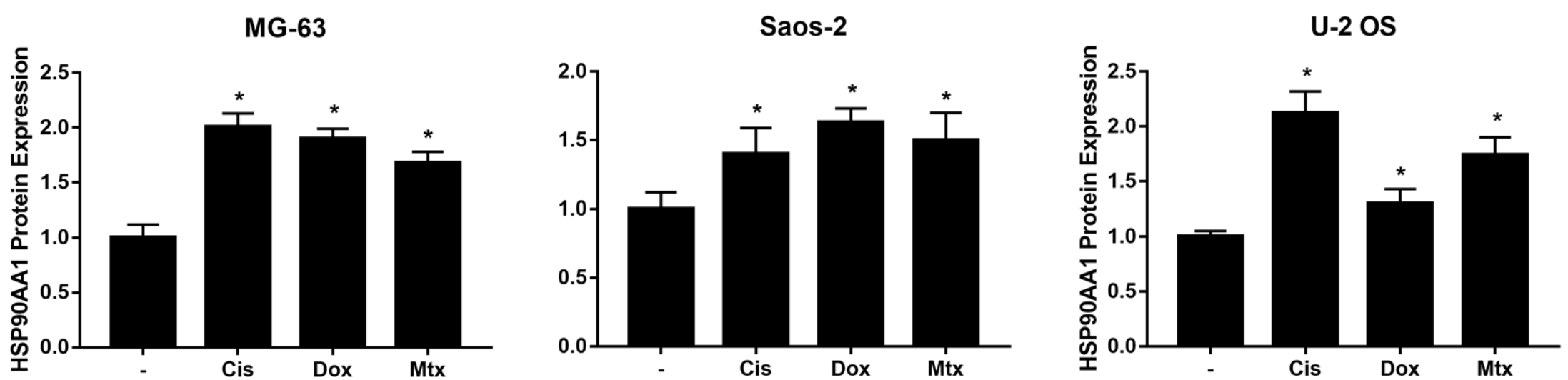

B

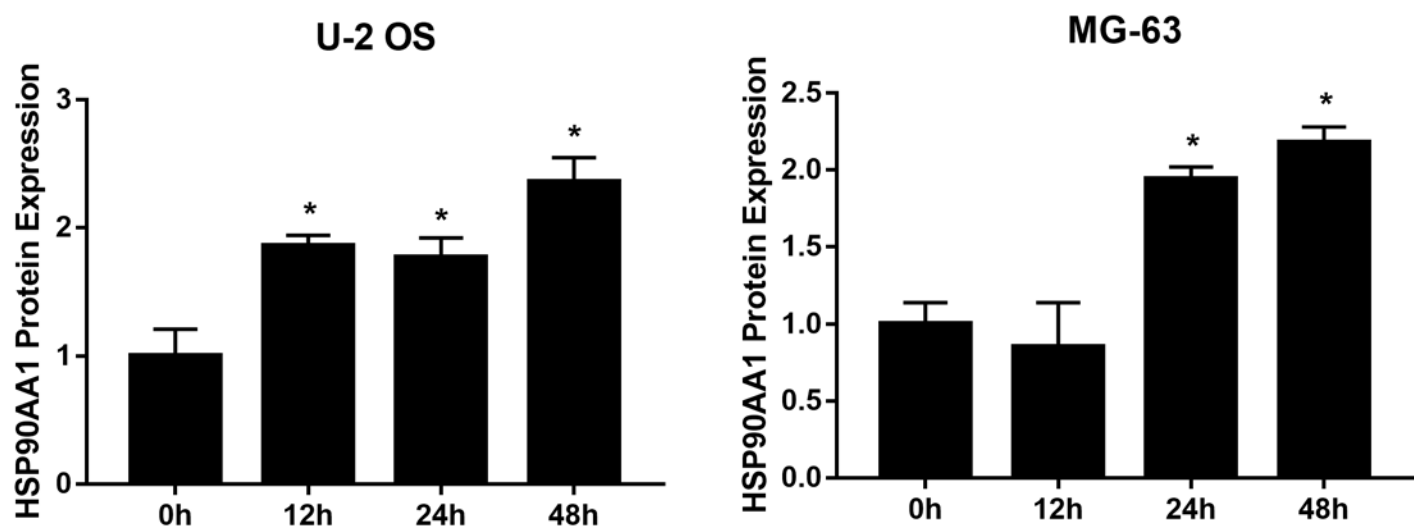

C

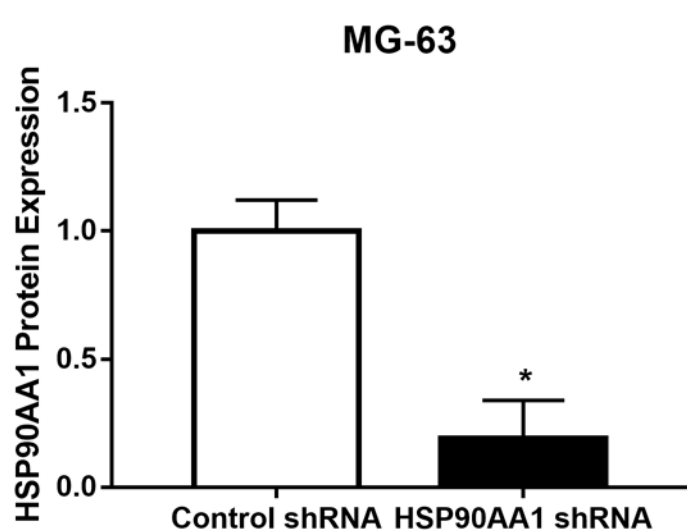

D

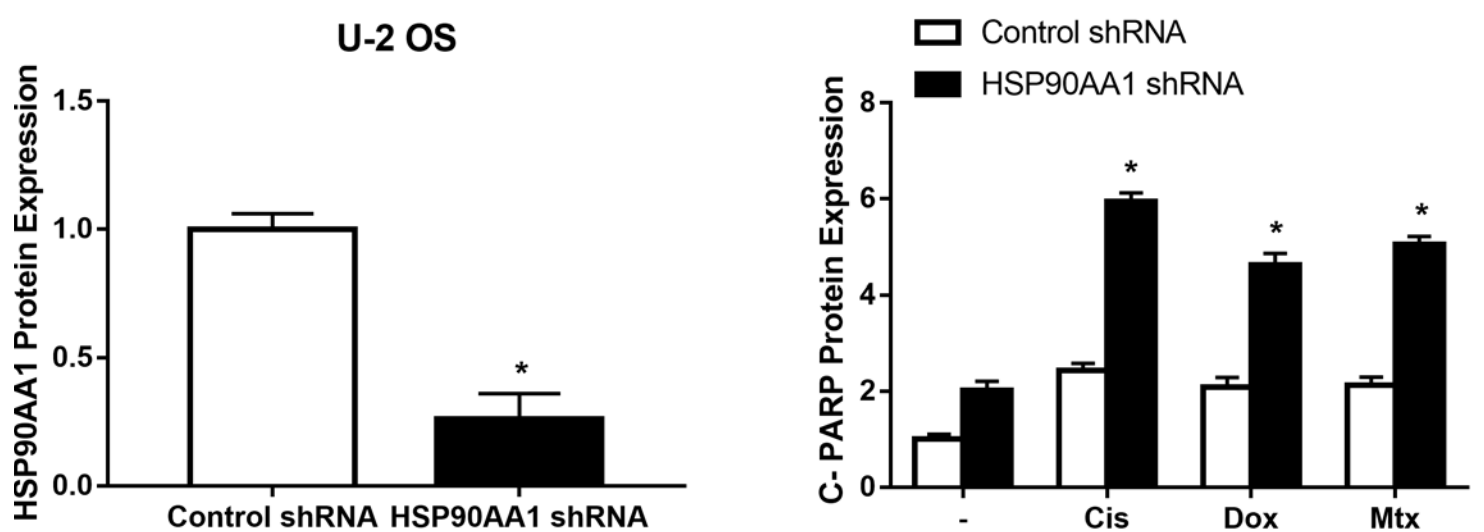

E

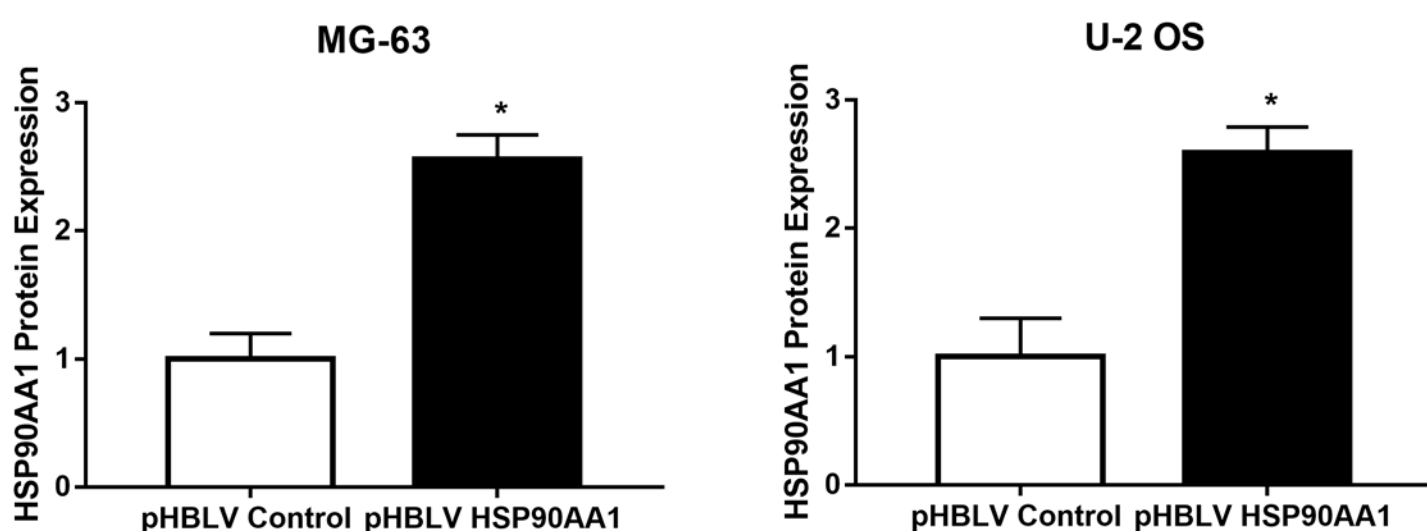

F

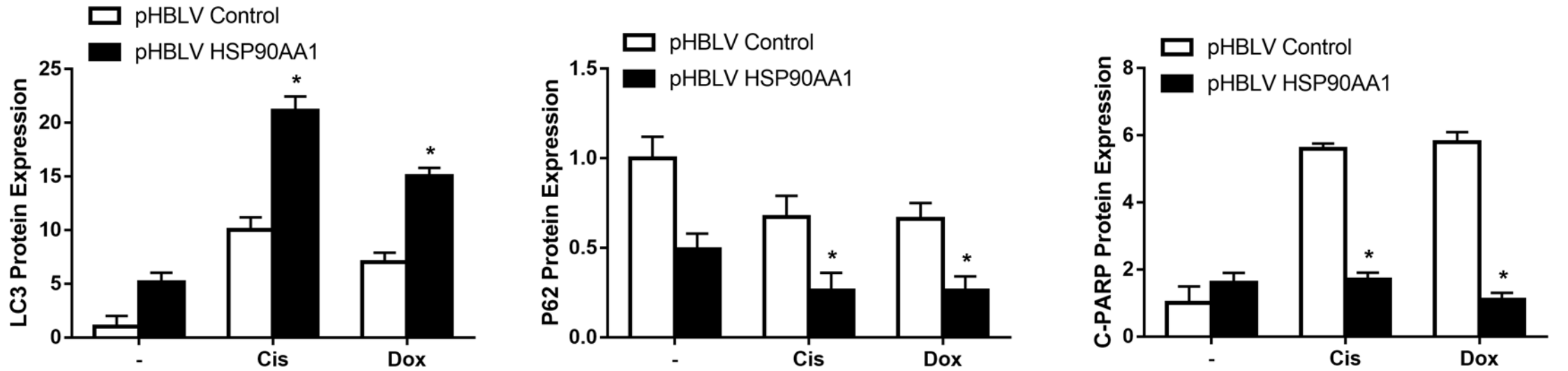

G

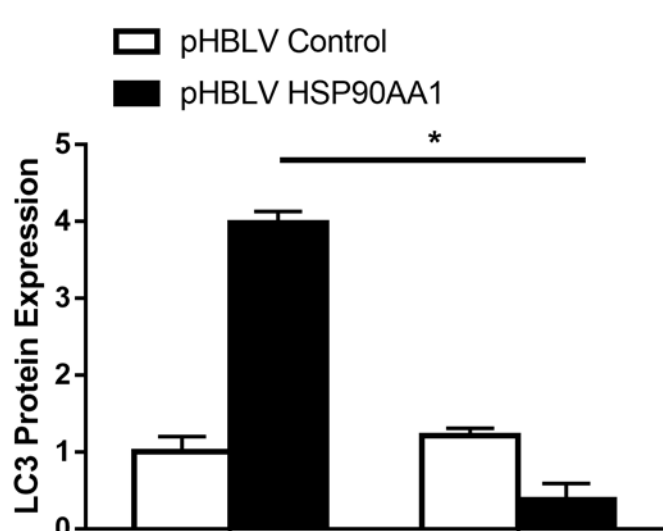

H

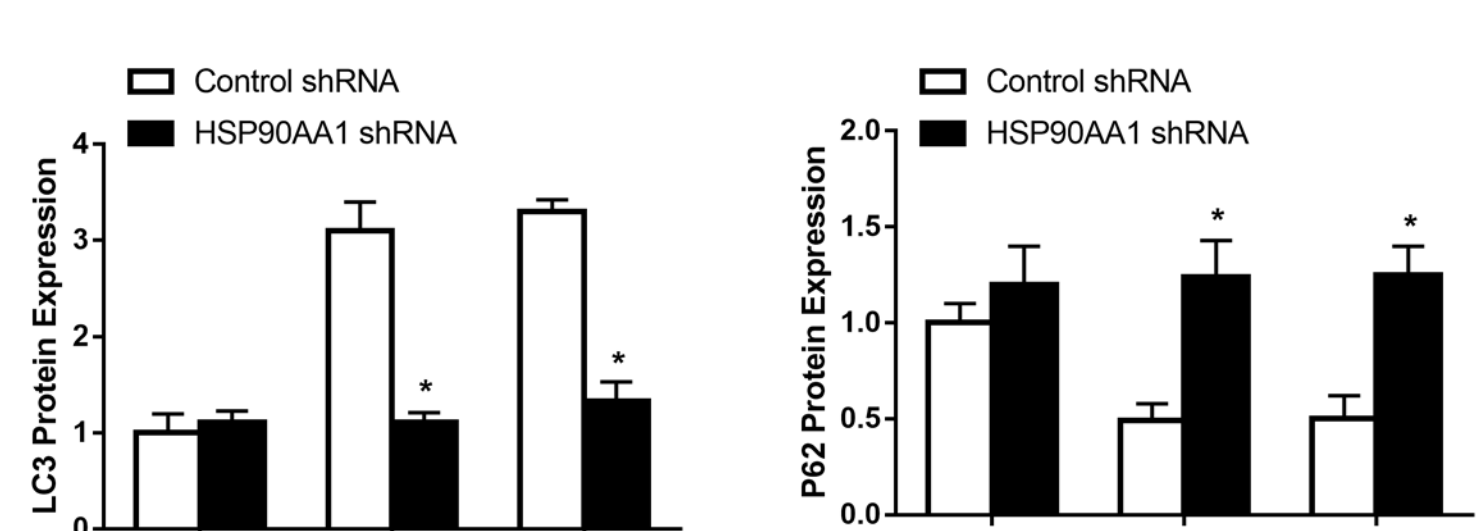

I

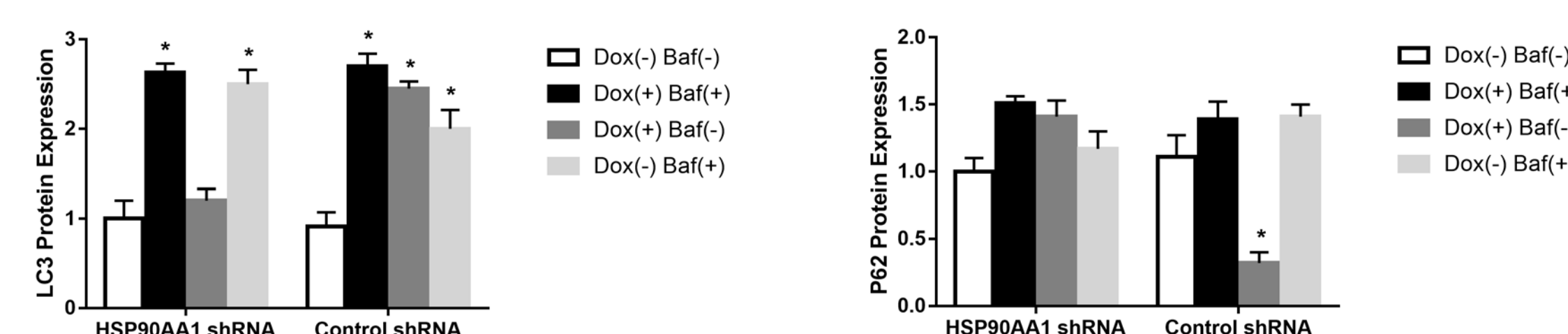

J

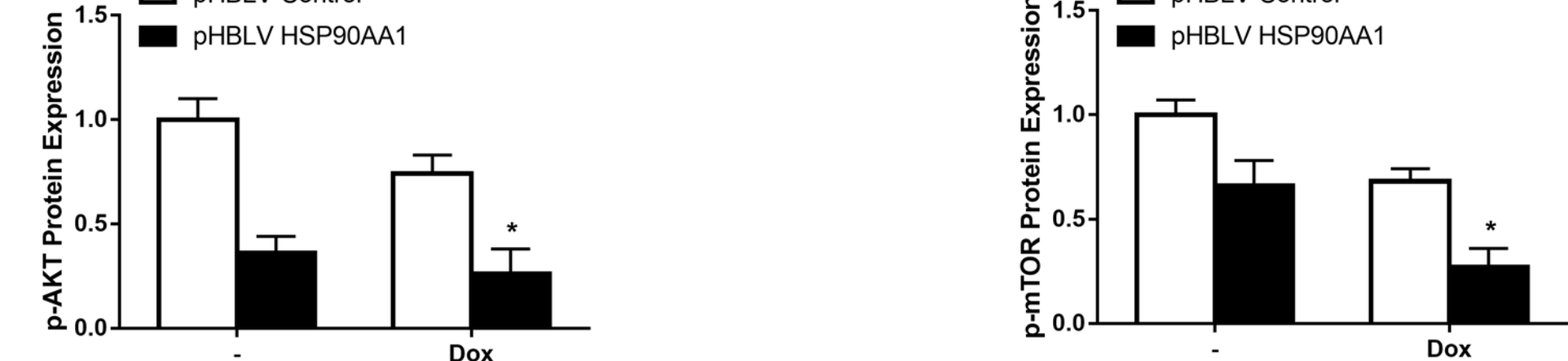

K

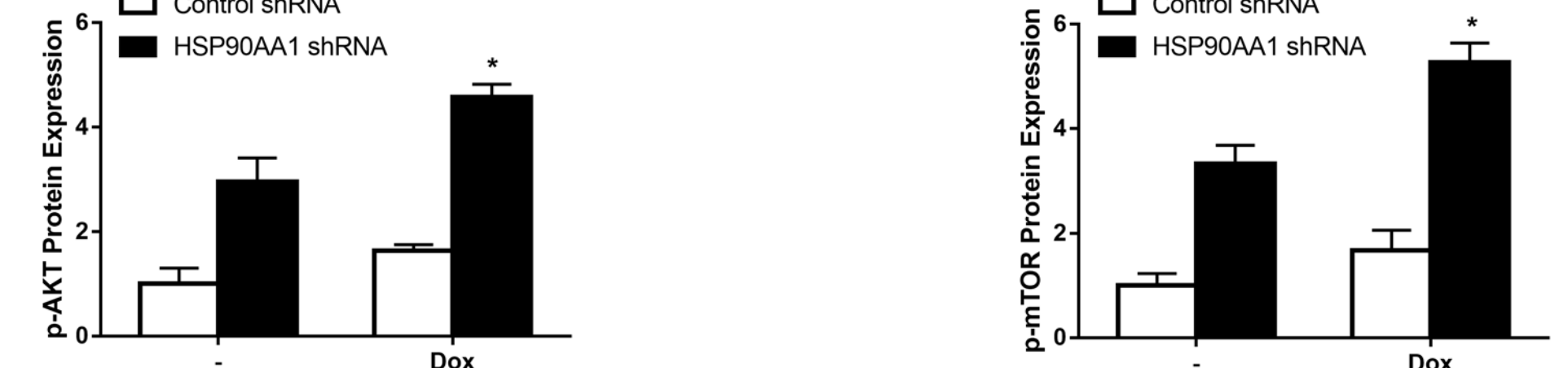

L

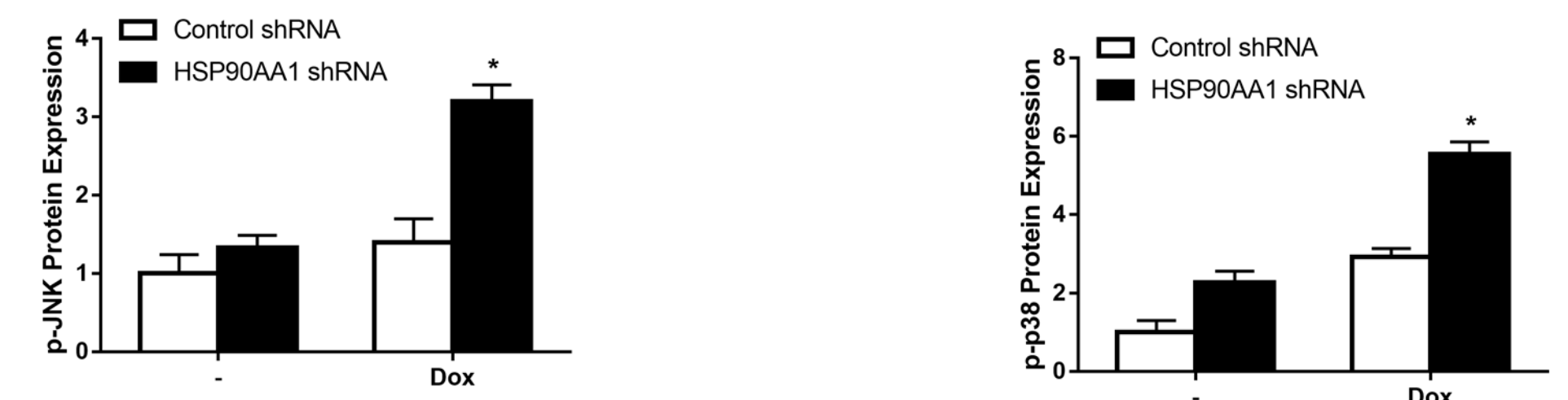

Supplement: Supplementary file 1 — Figure S1. Normalized quantification of all immunoblots in the manuscript. A, The normalized quantification of immunoblots in Fig. 1a. B, The normalized quantification of immunoblots in Fig. 1c. C, The normalized quantification of immunoblots in Fig. 2a. D, The normalized quantification of immunoblots in Fig. 2d. E, The normalized quantification of immunoblots in Fig. 2f. F, The normalized quantification of immunoblots in Fig. 3a. G, The normalized quantification of immunoblots in Fig. 3c. H, The normalized quantification of immunoblots in Fig. 4a. I, The normalized quantification of immunoblots in Fig. 4b. J, The normalized quantification of immunoblots in Fig. 5A. K, The normalized quantification of immunoblots in Fig. 5b. L, The normalized quantification of immunoblots in Fig. 5d. (n = 3; *, p < 0.05 versus control group). (PDF 304 kb) [file 13046_2018_880_MOESM1_ESM.pdf]

**A**

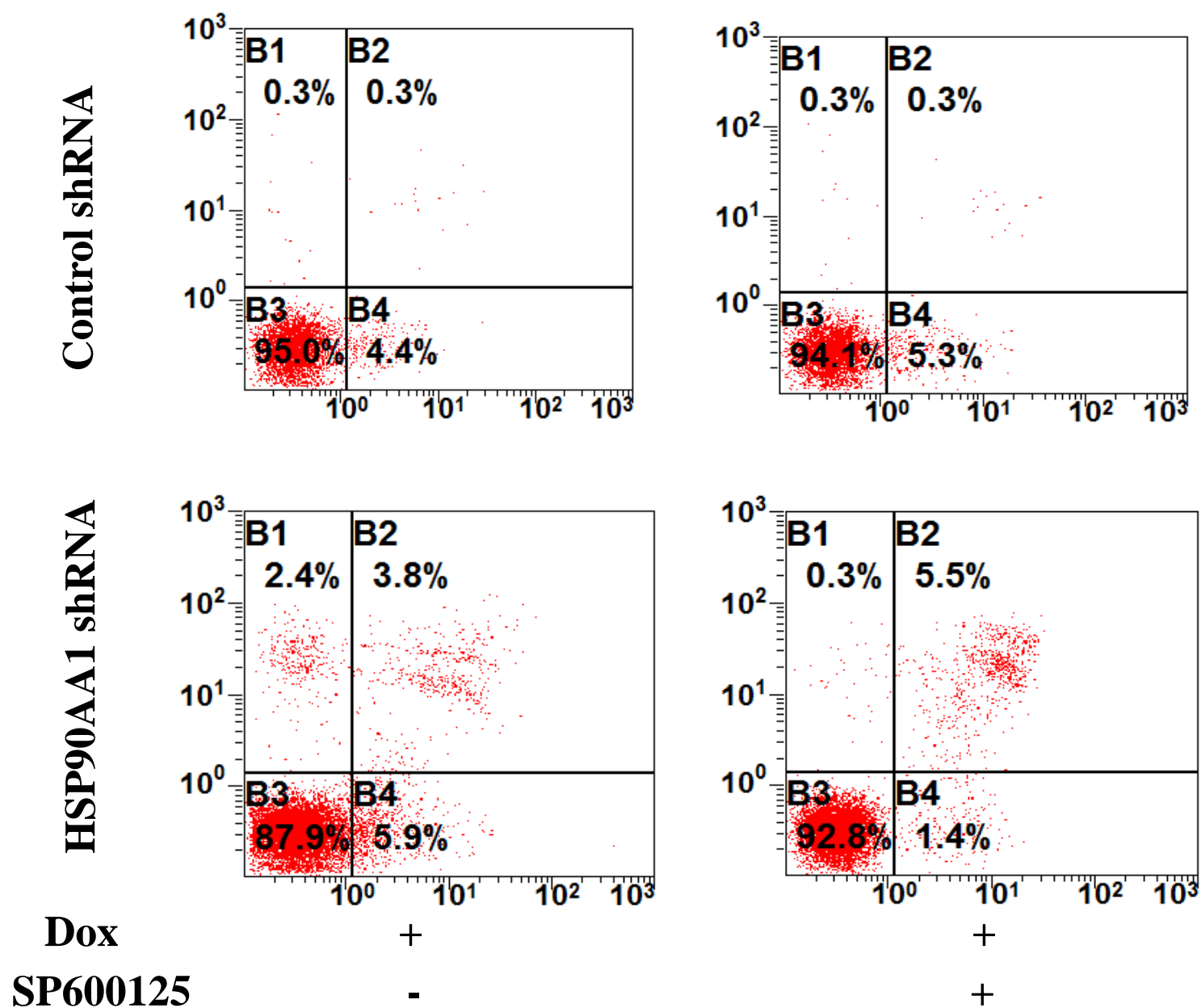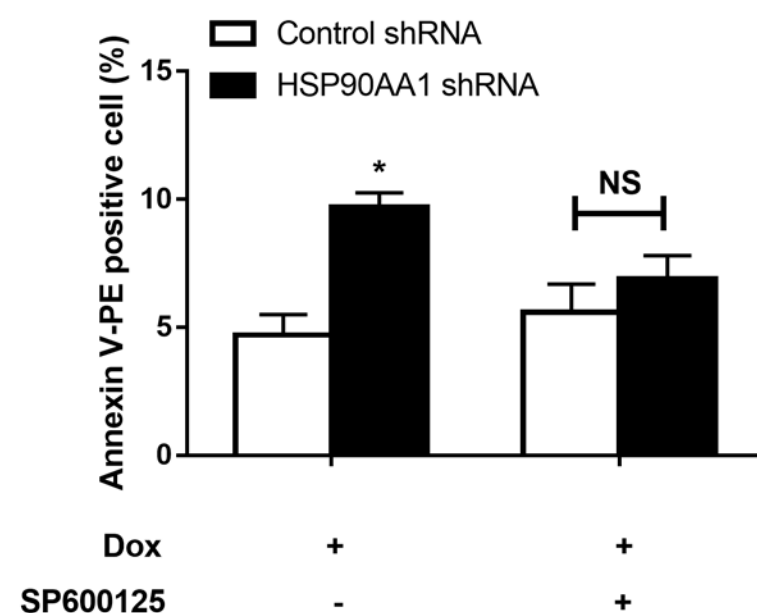

**B**

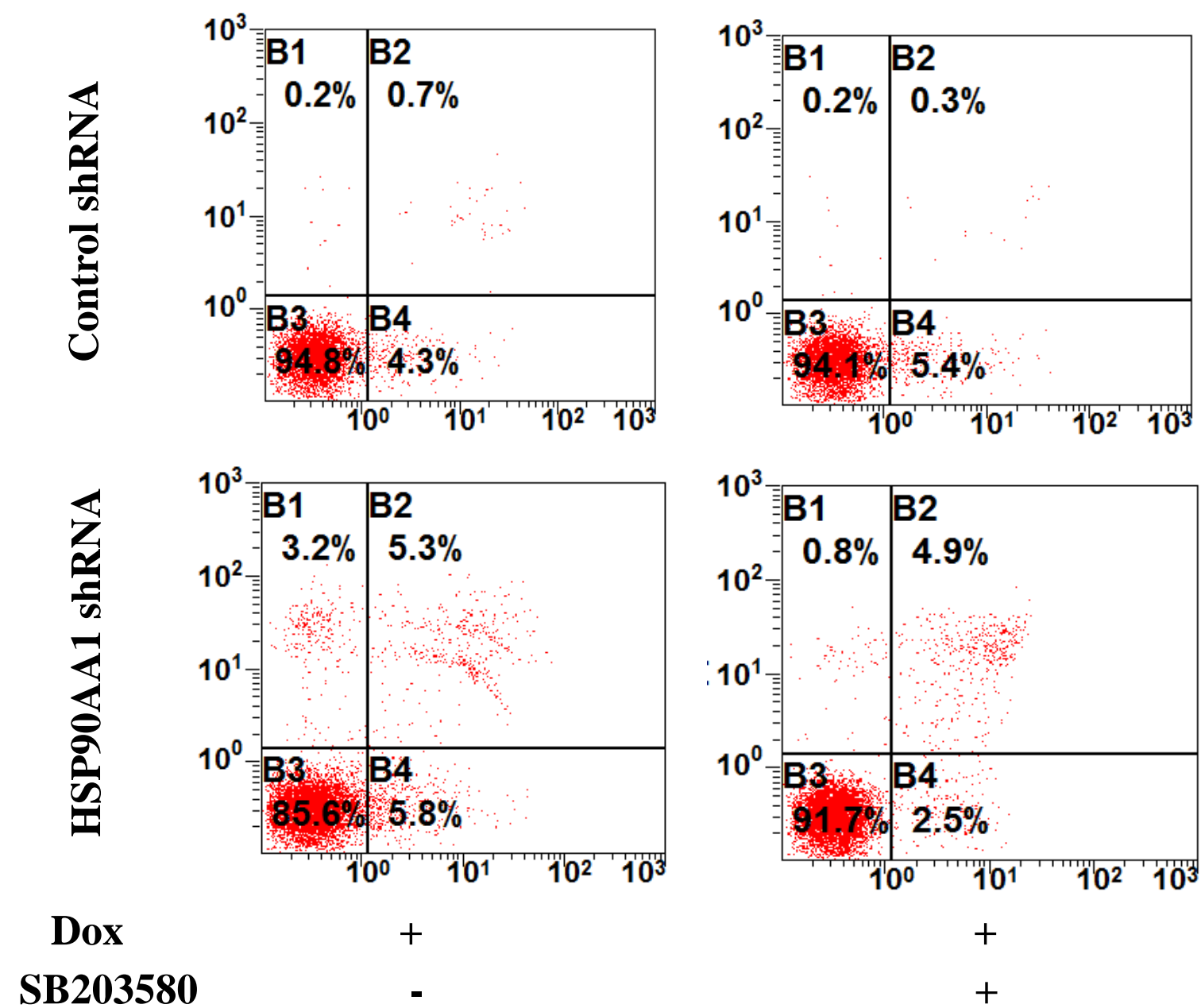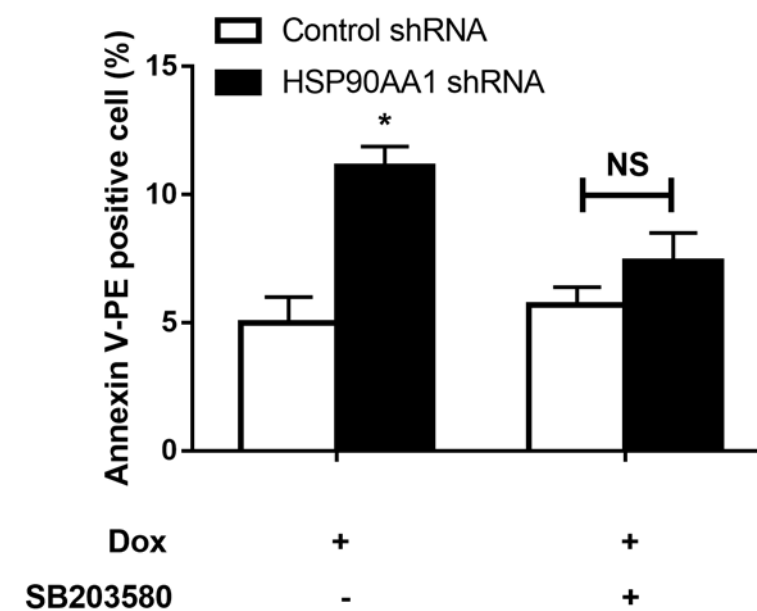

Supplement: Supplementary file 2 — Figure S2. JNK or p38 inhibitors decreased the cell apoptosis. A, control shRNA/HSP90AA1 shRNA transfected MG-63 cells were treated with Dox (0.2 μg/mL) for 24 h with or without SP600125. Apoptosis was analyzed by measuring Annexin V-PE/PI positive cells by flow cytometric (n = 3; *, p < 0.05 versus control shRNA group). B, control shRNA/HSP90AA1 shRNA transfected MG-63 cells were treated with Dox (0.2 μg/mL) for 24 h with or without SB203580. Apoptosis was analyzed by measuring Annexin V-PE/PI positive cells by flow cytometric (n = 3; *, p < 0.05 versus control shRNA group). NS, not significant. (PDF 88 kb) [file 13046_2018_880_MOESM2_ESM.pdf]
